# Bulwark Effect of Response in a Causal Model of Disruptive Clinician Behavior: A Quantitative Analysis of the Prevalence and Impact in Japanese General Hospitals

**DOI:** 10.3390/healthcare13050510

**Published:** 2025-02-26

**Authors:** Manabu Fujimoto, Mika Shimamura, Hiroaki Miyazaki

**Affiliations:** 1 Institute for Teaching and Learning, Ritsumeikan University, 56-1 Tojiin-Kitamachi, Kita-ku, Kyoto 603-8577, Japan; 2 Faculty of Nursing, Reiwa Health Sciences University, 2-1-12 Wajirogaoka, Higashi-ku, Fukuoka 811-0213, Japan; m.shimamura@rhs-u.ac.jp; 3 Medical Safety Management Center, Kansai Medical University, 2-5-1 Shinmachi, Hirakata-shi, Osaka 573-1010, Japan; miyazakh@hirakata.kmu.ac.jp

**Keywords:** disruptive clinician behavior, bulwark effect, psychological impact, hospital management impact, moderated mediation analysis, Japanese hospitals

## Abstract

**Background:** Disruptive clinician behavior (DCB) negatively affects patient safety by impairing healthcare team communication. In Japanese hospitals, hierarchical structures and traditional leadership styles contribute to its persistence. This study examines the prevalence and impact of DCB in two general hospitals and evaluates the role of response strategies in mitigating its effects. **Methods:** A quantitative web-based survey was conducted among 256 healthcare professionals from two general hospitals (751 and 661 beds). The survey included demographic data, a validated DCB scale, and a structured questionnaire assessing triggers, responses, and impacts. Statistical analyses included principal component analysis (PCA), structural equation modeling (SEM), and moderated mediation analysis. **Results:** Among participants, 79.3% reported experiencing or witnessing DCB. Psychological/social impact partially mediated the relationship between DCB and hospital management issues (*β* = 0.19, *p* = 0.001). Response strategies reduced the psychological/social impact of DCB (*β* = −0.20, *p* < 0.001) but did not mitigate its direct effect on hospital management. **Conclusions:** While prompt responses can alleviate the psychological burden on victims, they do not prevent broader institutional damage caused by DCB. Effective interventions should focus on both individual and organizational measures to reduce the occurrence of DCB.

## 1. Introduction

Disruptive clinician behavior (DCB) refers to unprofessional and unethical behavior that seriously affects patient safety by disrupting interpersonal relationships among healthcare providers and resulting in communication and teamwork dysfunction [1,2]. Disruptive interpersonal behaviors among healthcare providers harm communication and teamwork among providers, but the types of behaviors are diverse. A breakdown of disruptive behaviors showed physical violence accounted for less than 5%, but there were high incidences of abusive language, verbal abuse, rudeness, scolding, abusive anger, insults, and threats [3]. Other categories of such behaviors include sexual harassment, racism, threatening behavior, neglect, disrespect, throwing of items, and refusal to attend meetings [4,5]. The Disruptive Behavior Questionnaire (PASCAL METRICS, Inc., Washington, DC, USA) can be used to assess different categories of disruptive behaviors, including divisive, intimidating, disrespectful, inhibiting, and offensive behavior. Petrovic et al. identified the top 20 disruptive behaviors in a literature review, which they classified into eight categories: passive-aggressive behavior, physical or verbal threats, verbal abuse, physical violence, harassment, intimidation, bullying, and discrimination [6]. However, these categories have not been statistically substantiated. Fujimoto et al. developed a psychological scale based on free descriptions from witnesses and victims of DCB according to the scale construction procedure and confirmed its validity and reliability [7]. Those authors showed that DCB has a hierarchal structure and may be divided into interpersonal and job-related aggression. The former category, interpersonal aggression, consists of psychological aggression, incivility, ignoring, and physical violence. Psychological aggression was further subdivided into intimidation, reproof, threats, and abusive language. Job-related aggression comprises mismanagement practices and passive aggression. Mismanagement practices were further divided into non-supportive coercion and arbitrary decisions. Table 1 summarizes the hierarchical structure of DCB, categorizing interpersonal aggression (e.g., intimidation, abusive language) and job-related aggression (e.g., mismanagement, passive aggression), which are further subdivided based on observed behavioral patterns.

DCB occurs routinely in healthcare settings [8]. Since 2000, DCB has been viewed as a problem in the US; the Joint Commission adopted a zero-tolerance policy and is actively working to eradicate DCB [9]. In contrast, awareness of DCB is low in Japan, and many people believe it is inevitable that they will be subjected to DCB [10]. In this context, problems with the causal leadership system and hierarchy have been highlighted. The same is true in the US, where not all DCB is perpetrated with clear intent to harm. There is a small number of healthcare providers who unknowingly engage in violent and often angry behavior that affects interpersonal relationships and the quality of healthcare [8,11,12]. One of the few empirical studies focused on DCB in Japan found that this behavior often included explicit and spiteful behavior [13]. Explicit DCB may vent anger or frustration, expressed as physical violence or psychological aggression. Concerningly, organizations tend to overlook physicians at the top of the professional hierarchy, even when they engage in explicit DCB [8,14]. Spiteful DCB is tantamount to bullying in the workplace and often takes the form of incivility and ignoring. Many previous studies indicated that DCB among nurses, who make up the majority of healthcare workers, generally takes the form of bullying [15,16,17].

This study focused on Japanese healthcare providers. Well-known Japanese cultural characteristics include group orientation, sociability, and wholeness [18]. Furthermore, historically, Confucian morality has been solid, and hierarchical relationships are strict [19]. These characteristics mean that peer pressure and synchronized behavior are likely to occur, and members are unlikely to disagree with an organization’s norms and policies. Because of these cultural characteristics, Japanese organizations tend to be constitutionally psychologically insecure, which means that their members do not freely express opinions and ideas without fear of blame or criticism. This concept has recently been emphasized in the medical world [20], as organizations with higher levels of psychological safety show higher performance than those with lower levels of psychological safety [21]. Given that team medicine has become mainstream, it is assumed that if medical professionals are guaranteed an environment in which they can freely express their opinions, DCB will decrease, and the quality and safety of medical care will improve. It has been noted that Japanese society is feudalistic; however, other countries are similar, especially in medical institutions [22]. The shared cause of saving patients’ lives fosters an atmosphere in which anything is acceptable. A study focused on Japanese medical professionals is expected to provide essential insights in elucidating the reality of DCB that occurs in medical organizations that tend to be closed.

Based on the above research background, this study attempted to elucidate a causal model of DCB in the Japanese medical field. The Johns Hopkins model for DCB has four serially connected components: triggers, DCB, response, and impact [23]. Demonstrating the relevance of these components was one of the main tasks of our study. Regarding the model components, the impact of DCB has adverse effects on both the organization [24,25] and the victim [26,27]. Therefore, we treated impact discriminately. In addition, knowing how to respond when DCB occurs is essential [28,29]. For example, Rosenstein recommended a proactive approach to early detection and intervention and improving organizational culture because of the complexity of the nature, causes, and effects of disruptive behavior [4]. Therefore, this study focused on the effects of response to DCB in validating the model.

There are existing scales for measuring DCB [7]. In this study, we collected information about triggers, responses, and impact using items in an open-ended questionnaire that was organized and categorized. To examine the causal relationships within DCB, we tested an analytical model of causality. Additionally, an adjusted mediation model was employed to clarify the response effect within the analytical framework. The difference between this analytical model and the Johns Hopkins model is that the statistical analysis treated “response” as an adaptation variable. First, a direct path was set for the triggers-DCB-negative impact on the organization, with reference to the Johns Hopkins DCB model. Next, we added an indirect path to the DCB-negative impact on the organization that mediated the negative impact on the victim because DCB has a serious impact on patient safety by disrupting healthcare providers’ relationships and making communication and teamwork dysfunctional. Finally, to apply the response component in the Johns Hopkins model to our analytical model, arrows representing the response were drawn on the paths from DCB to the two impacts and between the two impacts.

Disruptive clinician behavior (DCB) negatively affects patient safety by impairing healthcare team communication. While previous studies have examined the impact of DCB, little is known about how responses from surrounding individuals can mitigate its effects. This study investigates the prevalence and impact of DCB and introduces the concept of the Bulwark Effect, in which appropriate responses from peers can alleviate the psychological and social impact on victims while leaving organizational and team-related consequences unaddressed.

This study aims to quantitatively analyze the prevalence and impact of DCB in Japanese hospitals, with a focus on how response strategies mitigate its effects. Specifically, we seek to (1) identify the key triggers of DCB, (2) evaluate its impact on healthcare management and psychological well-being, and (3) assess the effectiveness of response strategies through statistical modeling. These objectives align with our methodological framework, ensuring measurable and actionable insights.

## 2. Materials and Methods

### 2.1. Preliminary Survey

Participants included 712 nursing professionals nationwide enrolled in a survey panel of healthcare professionals owned by MyVoice, a web-based research firm. An open-ended Internet-based questionnaire was administered to them regarding their experiences of witnessing or being victimized by DCBs. The questionnaire items covered DCB, triggers, response, and impact. The responses regarding behavior were drawn from the “Psychological Scale for Measuring DCB” [7]. The descriptions collected for triggers, responses, and impacts were first summarized and then categorized using content analysis. Next, the responses were finalized after modification by the co-authors, who had significant experience in medical safety. In this process, triggers were categorized into 6 indicators and 12 items, and responses were categorized into 8 indicators and 16 items. The impact was dichotomized as a negative impact on either the organization or the victim. The former referred to the medical/managerial impact, which reflected the deterioration of the quality of medical care provided by the hospital and the hospital management. The latter comprised psychological/social impact, which referred to the deterioration of the psychological and social adaptation of the victims. Each of these impacts was classified using three indicators and six items.

### 2.2. Procedures and Participants

A field survey using Google Forms was administered to the staff of two general hospitals (751 and 661 beds). Documents outlining the survey’s purpose, methodology, and ethical considerations were distributed to the hospital staff. Staff members who read and agreed to the document could access the Google Forms questionnaire at their convenience by either entering the provided URL or scanning the QR code printed on the document. Among the 493 individuals who consented to participate in the survey (response rate: 16.38%), 258 reported experiencing victimization by DCB. Of these, two individuals did not consent to have their responses analyzed and were excluded. As a result, data from 256 participants were included in the final analysis. The demographic details of participants, including profession, gender, and age distribution, are summarized in Table 2.

### 2.3. Survey Items

First, participants were asked to respond to five items covering demographic information. Next, they completed the 38-item Psychological Scale for Measuring DCB, which comprises 10 indicators. The reliability and validity of this scale have been verified in previous research [7]. Participants were asked to rate the degree to which they had experienced the DCB in their case using a six-point scale (from “never” to “always”). Finally, participants completed the Inventory on Causal Variables of DCB, which comprehensively examined triggers, responses, psychological/social impact, and medical/managerial impact. The causal variables included in this inventory were collected in the preliminary survey. Responses were on a six-point scale (from “not at all” to “extremely”) to determine the degree of applicability. A full list of questionnaire items is provided in the Supplementary Materials (Table S1).

### 2.4. Study Design and Setting

This study employed a quantitative observational design to examine the prevalence and impact of disruptive clinician behavior (DCB) in Japanese hospitals. Data were collected through web-based questionnaire surveys conducted in two general hospitals.

The hospitals were selected from different large metropolitan areas in Japan. Among multiple candidate hospitals, these two were chosen based on their willingness to participate and their representative characteristics as large-scale general hospitals (751 and 661 beds, respectively).

### 2.5. Statistical Analyses

Multivariate analysis of variance was used to examine the differences between the data for the two hospitals. First, confirmatory factor analysis and calculation of the omega coefficient were used to check the reliability of the item sheets for the DCB causal variables. Next, principal component analysis was used to compress the factors for each indicator. Structural equation modeling (SEM) was then used to validate the direct path model. Furthermore, mediation analysis was used to examine the psychological/social impact of the mediation effects. Finally, moderated mediation analysis was used to test the moderation effect of a response. Statistical analyses were conducted using IBM SPSS Statistics (version 29.0.0.0) and IBM SPSS Amos (version 29.0.0).

### 2.6. Ethical Considerations

This study provided participants with a document explaining the research objectives, methods, and ethical considerations. Only those who gave their consent were allowed to participate in the survey. In the online survey, a consent confirmation screen was displayed before the questionnaire began, and only those who clicked the “I agree” button were able to proceed with their responses. All responses were collected anonymously, and no personally identifiable information (such as names, affiliations, or contact details) was obtained. The collected data were used solely for research purposes and were strictly managed. Additionally, the results were statistically processed and reported in a manner that ensured no individual respondent could be identified. This study adhered to relevant research ethics guidelines and was approved by the research ethics committees of Kansai Medical University Hospital (2023419) and JCHO Chukyo Hospital (2023021). In the preliminary survey, an email from a web research company was sent to panel registrants. Informed consent was obtained from the participants before they proceeded with the survey. In the main survey, after obtaining approval from the hospital administrators, a written request for research cooperation was distributed to hospital staff. These documents clearly stated the purpose and methods of the study, emphasized that participation was voluntary, and ensured that data would be statistically processed to maintain anonymity. Participants who agreed to cooperate in the study accessed the online questionnaire using the URL provided in the request document. The first page of the questionnaire contained the same information as the requested document. Participants who agreed to take part in the survey checked the consent option and then proceeded to complete the questionnaire.

## 3. Results

### 3.1. Confirmation of Differences Between Hospitals

To confirm the differences between data for the two hospitals, we performed a multivariate analysis of variance with the hospital as the independent variable and scale data as the dependent variables. The multivariate test was non-significant (Wilks’s L = 0.847, F(30, 225) = 1.358, *p* = 0.111, partial h2 = 0.153). Therefore, the data from the two hospitals were combined for the subsequent analyses.

### 3.2. Confirming the Reliability of the Indicators

We tested the reliability of the 20 indicators for the DCB causal variables in the item form. Each indicator had two items, and their respective omega coefficients ranged from 0.651 to 0.914, except for avoidance, which was low at 0.480 (Table 3). Confirmatory factor analysis was conducted after the scale scores were calculated. The results showed that triggers (goodness of fit index [GFI] = 0.965, comparative fit index [CFI] = 0.961, root mean square error of approximation [RMSEA] = 0.095), impact (GFI = 0.953, CFI = 0.979, RMSEA = 0.092), and response (GFI = 0.933, CFI = 0.962, RMSEA = 0.073) all showed a certain degree of fit. In terms of the impact, we used a model in which correlation paths were drawn between the observed variables representing the psychological/social impact and medical/managerial impact factors.

### 3.3. Compression of Indicators

We used principal component analysis to compress the indicators in the mediation and adaptation mediation analyses (Table 3). For DCB, nine indicators were highly loaded on the first principal component, except for physical violence, which had a significantly low mean value. For response, five indicators were highly loaded on the first principal component, except for three negative factors (avoidance, servile submission, and adulation). Therefore, “response” in the analytical model meant a positive response. All indicators for triggers, psychological/social impact, and medical/managerial impact were highly loaded on the first principal component. We, therefore, used the first principal component scores for all variables in the subsequent analyses.

### 3.4. Validation of DCB Triggers and Impact

**Figure 1:**
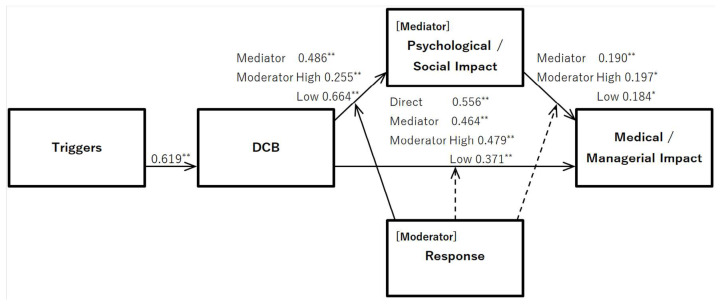
Causal model of DCB and estimated value of each path. Note: * *p* < 0.05, ** *p* < 0.01.

SEM was used to test the causal model of triggers-DCB-medical/managerial impact (Figure 1). The direct path from triggers to disruptive behavior and DCB to medical/managerial impact were significant, with estimates of 0.619 (standard error [SE] = 0.049, *p* < 0.001) and 0.556 (SE = 0.052, *p* < 0.001), respectively.

### 3.5. Examining the Indirect Effects of Psychological/Social Impact

We used mediation analysis to test the model, including indirect paths (Figure 1). The results showed that the direct path from DCB to medical/managerial impact had an estimated value of 0.464 (SE = 0.058, *p* < 0.001), the indirect path from DCB to psychological/social impact had an estimated value of 0.486 (SE = 0.055, *p* < 0.001), and the indirect path from psychological/social impact to medical/managerial impact had an estimated value of 0.190 (SE = 0.058, *p* = 0.001). To verify the mediating effect, we calculated 95% confidence intervals using the bootstrap method (sample size: 2000). The results were significant, with an estimate of 0.092 (SE = 0.032, *p* = 0.001; lower bound = 0.043, upper bound = 0.148). The partial mediation model was supported as the direct path estimate from DCB to medical/managerial impact was somewhat reduced from 0.556 to 0.464 by the mediation of psychological/social impact.

### 3.6. Verification of Moderate Effects of Response

We tested the moderating effect of response using a moderated mediation analysis (Figure 1). This analysis examined whether the effect of DCB on medical/managerial impact, mediated by psychological/social impact, was moderated by response. This approach allows us to determine whether the strength of the mediation effect varies depending on the level of response rather than assuming a uniform indirect effect.

First, we considered the paths for response. The path from response to medical/managerial impact was estimated at 0.125 (SE = 0.055, *p* = 0.025), and that from response to psychological/social impact was estimated at 0.133 (SE = 0.057, *p* = 0.020). The path from the interaction term between DCB and response to medical/managerial impact was estimated at 0.017 (SE = 0.046, *p* = 0.746), that from the interaction term between DCB and response to psychological/social impact was estimated at −0.204 (SE = 0.047, *p* < 0.001), and that from the interaction term between psychological/social impact and response to medical/managerial impact was estimated at 0.006 (SE = 0.051, *p* = 0.908).

Next, for the paths in the mediation models for DCB, medical/managerial impact, and psychological/social impact, the direct path from DCB to medical/managerial impact was 0.479 for the high response group (+1 standard deviation [SD]). The direct path from DCB to medical/managerial impact was 0.479 (SE = 0.071, *p* < 0.001). The indirect path from DCB to psychological/social impact was 0.255 (SE = 0.072, *p* < 0.001), the indirect path from psychological/social impact to medical/managerial impact was 0.479 (SE = 0.071, *p* < 0.001), and the indirect path from psychological/social impact to medical/managerial impact was 0.197 (SE = 0.078, *p* = 0.012). Conversely, for the low response group (−1 SD), the direct path from DCB to medical/managerial impact was 0.371 (SE = 0.087, *p* < 0.001), the indirect path from DCB to psychological/social impact was 0.664 (SE = 0.076), and the indirect path from psychological/social impact to medical/managerial impact was 0.184 (SE = 0.080, *p* = 0.023).

Finally, we tested the indirect effects using the bootstrap method. The results for the high response group (+1 SD) showed an estimated value of 0.055 (SE = 0.026, *p* = 0.034, 95% lower bound = 0.015, 95% upper bound = 0.121) and those for the low response group (−1 SD) showed an estimated value of 0.118 (SE = 0.053, *p* = 0.027, 95% lower bound = 0.016, 95% upper bound = 0.226), and were significant. Estimates for the direct path from DCB to medical/managerial impact were significantly lower in the low group (0.494–0.371) compared with the high group (0.528–0.479). Both the high and low groups supported the partial mediation model. However, the mediation effect was superior in the low group compared with the high group.

## 4. Discussion

This study aimed to investigate the prevalence of DCB in Japanese hospitals and assess its impact on victims’ psychological and social adaptation, as well as on healthcare quality and hospital management. Additionally, we examined the role of appropriate responses from colleagues in mitigating these effects using an analytical model to explore both direct and indirect effects.

Our findings indicate that personality traits and workplace climate issues contribute to disruptive behavior among medical personnel. DCB was found to have both direct and indirect negative effects: it directly impaired healthcare quality and hospital management while also indirectly endangering them by worsening the victim’s psychological and social adaptation (mediating effect).

Furthermore, our results support the Bulwark Effect, where appropriate responses from colleagues help protect the victim’s psychological and social adaptation (moderating effect of response). In particular, seeking social support and proactive problem-solving by colleagues were identified as effective strategies. However, the bulwark effect only mitigated the indirect impact through psychological/social adaptation. As a result, the direct negative effects of DCB on patient safety and hospital management remained unbuffered. In other words, the responses of the victims and those around them did not prevent adverse effects on medical and managerial conditions. In summary, there were two main findings from this study. The first finding was that it is crucial not to overlook DCB and ensure that responses to any incidents are appropriate to create an environment where medical personnel can continue to work with good well-being. However, regardless of the response, DCB damages the quality of hospital care and management. The second main finding was the need to prevent the occurrence of DCB to protect the quality of healthcare and management of the organization. Based on these findings, the following discussion focused on responding to and preventing DCB.

Interpersonal DCB can be dichotomized into overt and spiteful forms, but overt DCB, such as violence and verbal abuse, directly reduces the quality of healthcare [13]. These often occur as administrative sanctions in hierarchical relationships, educational reprimands in teaching relationships, or abuse from physicians to nursing staff/co-medicals as part of performing their duties as higher-ranking personnel [30]. Personal circumstances, such as the perpetrator’s personality, mood, or physical condition, may also be triggers [31]. Therefore, it is necessary to raise awareness so people understand that DCB is an “old idea”. Updating awareness is not limited to the perpetrators; the organizational culture must be improved as it is a problem for the entire organization to prevent DCB from being overlooked as “something that cannot be helped” if it is observed.

Explicit DCB tends to be more apparent, and violence and sexual crimes tend to be less common, as they may violate criminal law [13]. However, spiteful DCBs, such as bullying and neglect, tend to be overlooked [32]. Spiteful DCB worsens victims’ psychological and social adaptation status [33]. As a result, victims have difficulty performing their regular duties, which affects patient outcomes [34] or results in a decision to leave their jobs [35]. Furthermore, the quality of medical care and the management of the medical institution suffer significant damage [36]. As demonstrated in this study, protecting victims from the consequences of DCB can reduce the deterioration of their psychological and social adaptation status. As identified in the preliminary study (Table 2), specific responses are for the victim to cope directly or request instrumental and emotional support from those around them, who can act toward a solution or mediate between the victim and perpetrator.

Although the interpersonal aspect of DCB tends to be emphasized, this behavior also has a job-related aspect. Irresponsible personnel actions and unilateral decision enforcement by hospital organization managers that are unwanted by the parties involved may also be spiteful variants of DCB in that they are difficult to manifest. Poor management and guidance can reduce the victim’s work ethic [37]. In addition, the victim may engage in passive aggression [7], such as skipping medical appointments or refusing to perform to the best of their ability. The manager/leader will again reprimand and punish them for this explicit passive-aggressive work attitude. The victim then engages in more passive aggression. The main characteristic of job-related DCB is that the perpetrator and the victim switch places, creating a negative spiral. Breaking this chain is a critical issue for DCB prevention.

The findings of this study provide important implications for hospital management policies and workplace culture improvement. This study identified the “Bulwark Effect”, demonstrating that the psychological and social impact of DCB on victims can be mitigated by appropriate responses from those around them. Previous research has reported that DCB disrupts communication within healthcare teams and negatively affects the quality of care and patient safety [38]. Additionally, it has been suggested that ignoring DCB can exacerbate harm and foster a workplace culture that tolerates such behavior [8]. Therefore, hospital administrators should actively work to minimize the impact of DCB after its occurrence by promoting appropriate responses and strengthening support systems. This includes establishing response protocols and reporting systems for DCB incidents, as well as introducing mentoring programs and peer support systems. Furthermore, based on the principles of restorative justice [39], facilitating dialogues that involve the concerned parties can be an effective approach to addressing the issue. Rather than unilaterally restraining the perpetrator with traditional punishments (i.e., retributive justice), this discipline encourages dialogue among the perpetrator, victim, and other parties involved. It aims to rebuild interpersonal relationships, including with those involved (i.e., restorative justice). The more cohesive the group, the higher the performance [40]. Therefore, to improve the quality and safety of healthcare, the organization’s goal should be to create an environment in which all healthcare professionals have a tolerant mindset, build receptive relationships, and work with a high sense of well-being. Countermeasures against DCB can also be systematic DCB against perpetrators. It is important to remember that perpetrators are as much a part of the healthcare organization as the victims. Under the zero-tolerance principle adopted in the US medical safety measures, perpetrators are punished severely to deter further problems. However, this entails similar structural problems of job-related DCB, and the system of sanctions undermines trust and cooperation within the organization [41]. In a zero-tolerance approach, once a DCB occurs, the perpetrator becomes psychologically demotivated as their commitment to the organization and motivation for their job diminishes.

This study has several limitations. First, the data were collected through self-reported surveys at two hospitals, which may limit the generalizability of the findings. The results may not be directly applicable to hospitals with different cultural and organizational contexts. Additionally, since this study employed a quantitative observational design, it is not possible to establish strict causal relationships. Future research should adopt longitudinal studies to better clarify the causal links between DCB and its effects. Moreover, as this study relied on subjective psychological evaluation scales, response bias may have influenced the findings. To address this limitation, future studies should incorporate objective indicators such as workplace observations, third-party evaluations, and patient safety metrics. Finally, this study did not fully examine the long-term impact of appropriate responses to DCB on organizational culture and patient care quality. Future research should conduct intervention studies to evaluate the real-world effectiveness of response strategies in healthcare settings.

## 5. Conclusions

This study tested a causal model of DCB and confirmed the partially mediating effects of psychological/social impact and the moderating effects of a positive response. In addition, the finding that DCB directly and indirectly negatively affects the quality and management of hospital healthcare was important. However, this study used subjective evaluation measures based on a psychological paradigm. To reinforce the findings of this study, complementary verification using objective indicators is required. Having proposed prompt responses to DCB and organizational climate reforms to prevent it, we would like to conduct a fieldwork study to create and test specific plans in hospitals.

## Figures and Tables

**Table 1:** Empirical categories of disruptive clinician behavior.

**Interpersonal aggression**		
	Ignoring ^a^	
	Incivility ^b^	
	Physical violence	
	Psychological aggression	
		Intimidation ^a^
		Abusive language ^b^
		Reproof
		Threat ^c^
**Job-related aggression**		
	Mismanagement practice	
		Non-supportive coercion ^c^
		Arbitrary decision
	Passive aggression	

^a^ Silent aggression pair; ^b^ Personal aggression pair; ^c^ Power harassment pair.

**Table 2:** Participants’ demographic data.

	Hospital A	Hospital B
Physician	20 (23.8%)	11 (6.4%)
Nursing professional	38 (45.2)	101 (58.7)
Paramedic	21 (25.0)	38 (22.1)
Non-medical professional	5 (6.0)	22 (12.8)
Female	48 (57.1)	123 (71.5)
Male	35 (41.7)	44 (25.6)
Unanswered	1 (1.2)	5 (2.9)
Age (years) 20s	14 (16.7)	21 (12.2)
30s	21 (25.0)	40 (23.3)
40s	25 (29.8)	51 (29.7)
50s	17 (20.0)	36 (20.9)
60s	5 (6.0)	16 (9.3)
Unanswered	2 (2.4)	8 (4.7)
Total	84	172

**Table 3:** Results of principal component analysis of disruptive clinician behavior, triggers, response, and impact.

					*M*	*SD*	*w*
DCB			1st principal component contribution rate 35.03%				0.933
	Ignoring		1st principal component loading	0.605	2.51	1.50	
	Incivility			0.751	3.25	1.57	
	Reproof			0.839	3.03	1.50	
	Threat			0.837	3.79	1.50	
	Intimidation			0.739	2.67	1.44	
	Abusive language			0.845	3.50	1.54	
	Physical violence			0.351	1.24	0.69	
	Non-supportive coercion			0.684	1.77	1.02	
	Arbitrary decision			0.810	2.30	1.22	
	Passive aggression			0.746	2.41	1.31	
Triggers			1st principal component contribution rate 35.03%				0.789
	Perpetrator competence		1st principal component loading	0.575	2.80	1.47	0.753
		The perpetrator lacked knowledge or experience in the job			2.44	1.45	
		The perpetrator lacked competence and aptitude for the job			3.17	1.80	
	Perpetrator’s personality		1st principal component loading	0.669	3.84	1.43	0.651
		The perpetrator had communication and socialization problems			4.28	1.59	
		The perpetrator had a lax and inconsiderate personality			3.40	1.74	
	Victim’s competence		1st principal component loading	0.477	2.64	1.29	0.859
		The victim lacked knowledge or experience in the job			2.75	1.44	
		The victim lacked competence or aptitude for the job			2.53	1.32	
	Victim’s personality		1st principal component loading	0.556	1.93	0.99	0.782
		The victim had problems with communication and socializing			2.16	1.21	
		The victim had a lax and inconsiderate personality			1.70	0.99	
	Press of business		1st principal component loading	0.518	3.42	1.76	0.878
		There was a regular shortage of staff at the workplace			3.46	1.89	
		Busy or urgent situation			3.38	1.84	
	Normalization		1st principal component loading	0.721	4.02	1.53	0.681
		There was no one around to blame			4.45	1.65	
		Disruptive behavior was the norm in the workplace			3.59	1.85	
Response			1st principal component contribution rate 34.91%				0.868
	Direct action by the victim		1st principal component loading	0.488	2.90	1.52	0.854
		The victim tried to solve the problem by confronting the perpetrator and the problem			2.95	1.61	
		The victim tried to clear up the perpetrator’s misunderstanding			2.85	1.64	
	Requested instrumental support		1st principal component loading	0.824	3.06	1.51	0.669
		The victim told others about the harm			3.24	1.81	
		The victim asked for help from others			2.88	1.70	
	Requested emotional support		1st principal component loading	0.764	3.73	1.53	0.784
		The victim talked to others			3.82	1.70	
		The victim got emotional support from others			3.64	1.68	
	Avoidance		1st principal component loading	0.156	3.90	1.42	0.480
		The victim distanced themself from the perpetrator			4.03	1.79	
		The victim tried not to care			3.78	1.72	
	Servile submission		1st principal component loading	−0.031	3.26	1.60	0.733
		The victim had no choice but to give up			3.58	1.83	
		The victim did what the perpetrator wanted them to do			2.94	1.79	
	Direct action by others		1st principal component loading	0.797	2.05	1.29	0.890
		Others systematically took remedial measures			2.05	1.37	
		Others tried to solve the problem by confronting the perpetrator and the problem			2.05	1.35	
	Arbitration		1st principal component loading	0.791	1.86	1.19	0.812
		Others intervened between the perpetrator and the victim and tried to mediate			1.92	1.37	
		Others pacified the perpetrator			1.81	1.24	
	Adulation		1st principal component loading	0.081	2.28	1.32	0.782
		Others did what the perpetrator wanted them to do			2.60	1.57	
		Others sympathized with the perpetrator			1.95	1.36	
Psychological/social impact			1st principal component contribution rate 80.33%				0.923
	Psychological state		1st principal component loading	0.919	4.13	1.52	0.801
		Became physically and mentally unwell			3.99	1.69	
		Lost motivation to work			4.26	1.65	
	Quality of care		1st principal component loading	0.852	3.00	1.61	0.789
		Became confused and unable to think calmly			3.29	1.83	
		Impaired ability to provide medical care			2.70	1.73	
	Interpersonal relationships		1st principal component loading	0.917	3.96	1.51	0.755
		Communication became difficult			4.54	1.52	
		Difficulty staying in the workplace			3.39	1.84	
Medical/managerial impact			1st principal component contribution rate 69.47%				0.875
	Quality of medical care		1st principal component loading	0.892	3.31	1.62	0.768
		The workload increased because of staff shortages			3.09	1.83	
		Hindered the organization’s improvement in medical quality and safety			3.53	1.78	
	Hospital management		1st principal component loading	0.785	2.36	1.34	0.749
		Damaged the hospital’s social credibility and reputation			2.66	1.68	
		Caused financial damage to hospital management			2.05	1.34	
	Workplace relations		1st principal component loading	0.820	3.90	1.61	0.914
		Deteriorated the workplace atmosphere			4.06	1.66	
		Workplace teamwork deteriorated			3.75	1.69	

DCB = disruptive clinician behavior, *M* = mean, *SD* = standard deviation, *w* = omega coefficient.

## Data Availability

The raw data supporting the conclusions of this article will be made available by the authors upon request.

## Supplementary Materials

The following supporting information can be downloaded at https://www.mdpi.com/article/10.3390/healthcare13050510/s1, Table S1: Questionnaire Items.

## Abbreviations

The following abbreviations are used in this manuscript:

DCB	Disruptive clinician behavior
