# Supplementary material for: Bulwark Effect of Response in a Causal Model of Disruptive Clinician Behavior: A Quantitative Analysis of the Prevalence and Impact in Japanese General Hospitals
Source: Healthcare (Basel). 2025 Feb 26;13(5):510. doi: 10.3390/healthcare13050510 (PMC11899433; doi:10.3390/healthcare13050510)
Supplement: Supplementary file 1 [file healthcare-13-00510-s001.zip › healthcare-3472889-supplementary.pdf]

Supplementary Materials

Table S1. Questionnaire Items

	**Psychological Scale for Measuring DCB**<br>Please rate the following items on a six-point scale based on the extent to which they occurred.<br>(1: never, 2: rarely, 3: occasionally, 4: often, 5: frequently, 6: always)	Point
1	Talked behind others' backs	
2	Said things sarcastically	
3	Spoke ill of others	
4	Submitted others to derision	
5	Made no response	
6	Hung up the phone during a conversation	
7	Ignored others	
8	Behaved insensitively	
9	Behaved in a discriminatory manner	
10	Talked abusively	
11	Vented their anger on someone or something	
12	Nudged with the hand or foot	
13	Threw objects at someone	
14	Used violence against others	
15	Expressed discomfort or disgust	
16	Glared at others	
17	Overawed others	
18	Yelled at others	
19	Threatened others	
20	Neglected their duties	
21	Adopted an uncooperative posture	
22	Contravened an overall agreement	
23	Provided inadequate treatment for the patient	
24	Did not give the necessary information	
25	Gave inappropriate and unreasonable instructions	
26	Set policies without any explanation or consultation	
27	Did not provide the required guidance	
28	Convened unnecessarily	
29	Provided inappropriate guidance	
30	Talked harshly	
31	Gave others a reprimand	
32	Criticized others in public	
33	Persistently scrutinized mistakes	
34	Denied others' opinions unilaterally	
35	Enforced participation in fraud	
36	Coerced others into doing unreasonable work	
37	Did not provide the necessary work support	
38	Did not assign other jobs	

	**Trigger**<br>Please rate the following items on a six-point scale based on how much they applied to you.<br>(1: not at all, 2: barely, 3: slightly, 4: somewhat, 5: quite, 6: extremely)	Point
	**Perpetrator's Competence**	
1	The perpetrator lacked the necessary knowledge and experience for their job.	
2	The perpetrator lacked the required competence and aptitude for their job.	
	**Perpetrator's Personality**	

3	The perpetrator had difficulties with communication and interpersonal relationships.	
4	The perpetrator was irresponsible and inconsiderate.	
	**Victim's Competence**	
5	The victim (you) lacked the necessary knowledge and experience for your job.	
6	The victim (you) lacked the required competence and aptitude for your job.	
	**Victim's Personality**	
7	The victim (you) had difficulties with communication and interpersonal relationships.	
8	The victim (you) was irresponsible and inconsiderate.	
	**Work Overload**	
9	There was a chronic shortage of staff in the workplace.	
10	The workplace was under constant pressure and a heavy workload.	
	**Entrenched State**	
11	There was no one around to address or intervene in misconduct.	
12	Destructive behavior had become normalized in the workplace.	
	**Response**<br>Please rate the following items on a six-point scale based on how much they applied to you.<br>(1: not at all, 2: barely, 3: slightly, 4: somewhat, 5: quite, 6: extremely)	Point
	**Direct Resolution**	
1	The victim (you) confronted the perpetrator or the problem to resolve it.	
2	The victim (you) attempted to clear up the perpetrator's misunderstanding.	
	**Instrumental Social Support**	
3	The victim (you) reported the harm to workplace personnel.	
4	The victim (you) received help from workplace personnel.	
	**Emotional Social Support**	
5	The victim (you) consulted with workplace personnel.	
6	The victim (you) received emotional support from workplace personnel.	
	**Avoidance**	
7	The victim (you) distanced yourself from the perpetrator.	
8	The victim (you) tried to ignore the situation.	
	**Submission**	
9	The victim (you) endured the situation without taking action.	
10	The victim (you) complied with the perpetrator's demands.	
	**Workplace Personnel's Intervention**	
11	Workplace personnel implemented organizational improvements.	
12	Workplace personnel confronted the perpetrator or the problem to resolve it.	
	**Workplace Personnel's Mediation**	
13	Workplace personnel intervened between the perpetrator and the victim (you) to mediate.	
14	Workplace personnel attempted to calm the perpetrator.	
	**Workplace Personnel's Compliance**	
15	Workplace personnel complied with the perpetrator's demands.	
16	Workplace personnel sided with the perpetrator.	
	**Psychological / Social Impact**<br>Please rate the following items on a six-point scale based on how much they applied to you.<br>(1: not at all, 2: barely, 3: slightly, 4: somewhat, 5: quite, 6: extremely)	Point
	**Psychological State**	
1	I experienced physical and/or mental distress.	
2	I lost motivation for work.	
	**Interpersonal Relationships**	
3	I found communication more difficult.	

4	I felt uncomfortable staying in the workplace.	
	**Medical Care**	
5	I became confused and was unable to think calmly.	
6	I had difficulty performing medical care due to psychological distress.	

**Medical / Managerial Impact**<br>Please rate the following items on a six-point scale based on how much they applied to you.<br>(1: not at all, 2: barely, 3: slightly, 4: somewhat, 5: quite, 6: extremely)		Point
	**Workplace Relationships**	
1	The workplace atmosphere deteriorated.	
2	Teamwork in the workplace worsened.	
	**Organizational Healthcare**	
3	Staff shortages led to an increased workload.	
4	The improvement of healthcare quality and safety in the organization was hindered.	
	**Organizational Management**	
5	The hospital's social credibility and reputation were damaged.	
6	The hospital suffered financial losses.	
